# Prevalence and socio-economic determinants of growth and developmental delays among Iranian children aged under five years: A cross sectional study

**DOI:** 10.1186/s12887-024-04880-2

**Published:** 2024-06-26

**Authors:** Mehran Alijanzadeh, Nilofar RajabiMajd, Masoumeh RezaeiNiaraki, Mark D. Griffiths, Zainab Alimoradi

**Affiliations:** 1 Social Determinants of Health Research Center, Research Institute for prevention of Non- Communicable Diseases, Qazvin University of Medical Sciences, Qazvin, Iran; 2 International Gaming Research Unit, Psychology Department, Nottingham Trent University, Nottingham, UK

**Keywords:** Social determinants of health, Growth and development delays, Children, Iran

## Abstract

**Background:**

The main cause of growth and development delays remains unknown, but it can occur as an interaction between genetic, environmental, and socio-economic factors.

**Objective:**

The aim of the study was to investigate the prevalence and social determinants of growth and developmental delays among children aged under five years in Qazvin, Iran.

**Methods:**

A cross-sectional study was conducted between January 2019 to December 2020 with participation of 1800 mothers with children aged 4–60 months who were referred to comprehensive health centers in Qazvin city, Iran. Structural and intermediate social determinants of health were assessed including: parents and children socio-demographic characteristics, families’ living and economic status, parents’ behavioral factors, household food security, mother’s general health, and perceived social support. Children’s growth was assessed based on their anthropometric assessment and their development was assessed using their age-specific Ages and Stages Questionnaire. Data were analyzed using univariable and multivariable logistic regression models using SPSS software version 24 and Stata version 14.

**Results:**

The prevalence of developmental problems in each domain were 4.28% for personal and social delay, 5.72% for gross motor delay, 6.5% for communication delay, 6.72% for fine motor delay, and 8% for problem-solving delay. The prevalence of weight growth delays was 13.56% and height growth delays was 4.66%. Communication, gross motor, and problem-solving delays were higher among children whose fathers’ smoked cigarettes. Fine motor delays were lower among mothers with education status of high school diploma and university degree vs. the under diploma group. Personal and social delay was significantly higher among families with fair economic status and lower among children when their fathers were employed (vs. unemployed). Weight and height growth delays were higher among mothers who had experienced pregnancy complications and household food insecure families, respectively.

**Conclusion:**

There are different predictors of growth and developmental delay problems among Iranian children aged under five years including fathers’ smoking, families’ economic status, and household food insecurity as well as history of mothers’ pregnancy complications. The present study’s findings can be used to screen for at-risk of growth and developmental delays among children and could help in designing and implementation of timely interventions.

## Introduction

Growth and development is a continuous and complex process of acquiring various capabilities for optimal performance in the social environment, most of which occur in the first few years of life [1]. Five different developmental domains have been described comprising (i) communication, (ii) gross motor, (iii) fine motor, (iv) personal and social, and (v) problem-solving [2]. Age-specific milestones or skills are defined for each developmental domains, and if these milestones are not acquired, developmental delay occurs [1].

The main cause of developmental delay disorders remains unknown, but it can be influenced by social determinants and by experiencing inequities during the first eight years of life [3]. Social determinants of health comprise a wide spectrum of variables. In this regard, health systems need to identify the most influential social determinants of health in their systems to achieve justice in health by integrating proper screening and providing healthcare based on social risk factors and needs [4]. In previous studies, association of different SDH-related variables in relation to childhood growth and development have been investigated including governmental health policies [5, 6], economic crisis and poverty [7–9], food insecurity [10–12], parental unemployment [13, 14], and poor access to health services [15, 16]. Despite the wide range of social determinant factors, previous studies have investigated the influence of only a few SDH-related variables on children’s health.

The World Health Organization (WHO) has developed a conceptual framework to determine the relationships between determinants and their effects on health [17]. According to this conceptual framework, the social factors that determine health are: (i) *social, economic and political factors*, including government, political institutions and economic processes, culture and social system performance; (ii) *structural factors* (including education, income, gender, ethnicity, employment status) which lead to the creation of social and economic inequalities and ultimately the formation of social class; and (iii) *intermediate or mediating factors* which refer to the paths of the effect of structural factors on health. Intermediary factors include living environment conditions (place of residence, purchasing power, and work environment), psychosocial conditions (psychosocial stress, stressful life conditions and interpersonal relationships, stress control and social support), behavioral and biological factors (nutrition, physical activity), alcohol and tobacco consumption, genetic factors, and factors related to the health services delivery system [18].

Developmental delay disorders are among the most common problems of children. Globally, these delay disorders affect 180 to 200 million children aged under five years annually, with more than two-thirds occurring in low- or middle- income countries [2]. Also, it is estimated that 43% of children aged under 5 years in low- and middle-income countries (249.4 million individuals) are at risk of stunted growth [19]. In 2018, among children aged under 5 years globally, an estimated 7.3% had wasting growth (49 million) and an estimated 21.9% (149 million) had stunted growth [20]. In Iran (where the present study was carried out), different prevalence of growth and developmental delay had been reported to be between 3.69% and 18.8% [21–23]. Although previous studies have investigated the prevalence of growth and developmental delay disorders among Iranian children, the most recent studies were conducted more than five years ago which necessitate the need for updated studies. Also, to best of the present authors’ knowledge, no previous study has been conducted in Qazvin, Iran (the previous nationally representative study used quota sampling and did not include any more than 360 children from any one region [21]).

Moreover, despite the fact that conceptual framework of social determinants of health formulated by the World Health Organization was first introduced in 2010, it has not been properly used in previous studies to evaluate the relationship between social factors affecting health and children’s growth and development. Considering these gaps, the present study aimed to answer the fallowing research questions:

(i) What is the prevalence of growth and developmental delays among children aged under five years in Qazvin, Iran?
(ii) What are the social determinants of growth and development delays among children aged under five years in Qazvin, Iran?

## Methods

### Study design

A cross-sectional study was conducted between January 2019 and December 2020.

### Setting

Using convenience sampling, recruitment was carried out at all 15 comprehensive health centers in Qazvin city. A wide range of healthcare services including children’s growth and development monitoring and children’s vaccination program are provided in comprehensive health centers. Based on a report by Qazvin University of Medical Sciences’ health deputy, more than 85% of under five children utilize related health services in comprehensive health centers. This help researchers to reach participants with maximum variety of scio-demographic characteristics.

### Participants

Mothers with children aged 4–60 months who visited health centers in Qazvin city were eligible for inclusion if they (i) were of Iranian nationality, (ii) had the ability to read and write, and (iii) had a child aged 4–60 months with no history of chronic or congenital diseases, normal birth weight (2500 to 4000 g), and a normal term birth (gestational age 38 to 42 weeks at the time of birth). Potential participants were excluded if they had a child with a history of surgery or serious illness that led to hospitalization of the child, or they did not want to participate in the study.

### Sampling procedure

In each comprehensive health center, a list of names and contact number of mothers with a child aged 4–60 months were extracted. They were called and invited to participate in the study by explaining the study aims, and assured of the confidentiality of their data. An appointment was then arranged at the health center at a suitable time for the mothers to provide the child’s healthcare status and completing the study questions with the help of the interviewers. The interviewers were five trained healthcare providers (familiar with the objectives of the research). Their accuracy on measurements and interviews were ensured by principal investigator of research (for each interviewer, 10 sessions of anthropometric assessment and data collection was observed). The appointment time was set based on preference of participants. Each interview session took 45 to 60 min. A small gift was given to participants to acknowledge their help in completing the interview session.

### Variables

The WHO conceptual framework including structural and intermediate factors were selected for assessing social determinants of health as below.

- Parent-related information was asked including their age, education, and marital status of parents (living together, divorced, death of one parent, child living with another guardian).
- Child-related information was asked including their age, gender, birth order, birth weight, history of maternal complications during pregnancy, and number of children in the family.
- A question on living environment conditions (i.e., rural/urban place of residency) was asked.
- The family economic status was evaluated by assessing perceived family economic status, house ownership, having healthcare insurance, and parent employment status.
- Questions relating to behavioral factors were asked including parents’ cigarette smoking, alcohol use, and illicit drug use.

### Measurement

Food access was assessed using the Household Food Insecurity Access Scale (HFIAS). The HFIAS assesses changes in food quality based on household’s perception with nine items rated on a four-point scale from 0 (*rarely*) to 3 (*often*). Higher scores indicate greater food insecurity. Scores higher than 2 are considered to indicate food insecurity. The psychometric properties of original [24] and Persian [25] versions showed good validity and reliability. Cronbach’s α in the present study was 0.89.

Psychosocial conditions including mother’s general health and perceived social support were assessed using psychometric scales. The 28-item General Health Questionnaire (GHQ) [39] comprising four subscales (somatic symptoms, anxiety and sleeplessness, social dysfunction, and severe depression) was used to assess health. Items are rated on four-point scale from 0 (*better than usual*) to 3 (*much worse than usual*). Total scores range from 0 to 84, with higher scores indicating a greater possibility of pathological health symptoms [26]. The psychometric properties of the original [27] and Persian [28] versions showed good validity and reliability. Cronbach’s α in the present study was 0.82.

The 12-item Multidimensional Scale of Perceived Social Support (MSPSS) comprising three different sources of support (family, friends, and significant others) was used to assess perceived social support. Items are rated on a five-point scale from 1 (*completely disagree*) to 5 (*completely agree*). Total scores range from 12 to 60 with higher scores indicating higher perceived social support [29]. The psychometric properties of original [29] and Persian [30] versions showed good validity and reliability. Cronbach’s α in the present study was 0.92.

#### Anthropometric indices

Two anthropometric indicators of low height for age and low weight for age based on z-score <-2SD were used. First, the child’s height and weight were assessed using a standard scale. All of anthropometric measurements were done with adherence of standard measurement guideline [31]. Their height and weight were then compared to the national age- and gender-specific z-scores. If their height or weight were under − 2SD of the determined z-scores, they were classed as having weight and/or height growth delay [32]. Low weight-for-age is defined as being underweight and low height-for-age is defined as being stunted [33].

#### Developmental status

Developmental status was assessed using 19 different age-specific versions of the Ages and Stages Questionnaire (ASQ) for children of different ages (i.e., 4, 6, 8, 10, 12, 14, 16, 18, 20, 22, 24, 27, 30, 33, 36, 42, 48, 54 and 60 months old). All ASQs assess five domains of development including communication, ​​gross motor, fine motor, problem-solving, and personal-social skills using 30 questions (six questions for each domain) [34]. Questions are responded to as either “Yes” (scoring 10 points) when the child is completely able to perform the activity in question, “Not yet” (scoring 0 points) when the child has not performed the activity in question, or “Sometimes” (scoring 5 points) when the child has performed the activity in question at some point previously. Total score for each domain is the sum of items which is then compared with the predetermined national age specific cut-off points (scores under − 2SD are determined as developmental delay) [35]. The psychometric properties of original [34] and Persian versions [35] showed good validity and reliability. Cronbach’s α in the present study was 0.90.

#### Study size

Based on previous reported prevalence of developmental delay among Iranian children (3.69% to 4.31% in different developmental areas [21]), and considering *p* = 0.04, α = 0.05, *d* = 0.01, using below formula and 20% attrition, a sample size of 1770 participants (i.e., mothers with a child aged under five years) was recommended.

$$n = \frac{{{z^2}_{\left( {1 - \frac{\alpha }{2}} \right)}P\left( {1 - P} \right)}}{{{d^2}}}$$

#### Statistical methods

Data were analyzed using SPSS version 24 (IBM crop, New York, USA) and Stata version 14. Continuous data were summarized using means and standard deviations (SDs), and categorical variables were presented using frequencies and percentages. Univariable and multivariable logistic regression was conducted. In the logistic regression method, the response variable should be the two-state qualitative type. In the present study, the response variable was defined as whether the child had the delay or not in each domain of growth and development. Qualitative variables with categorical rating scales were defined as dummy variables before implementing the model. Also, multi-collinearity was detected based on independent variables inter-correlations. To identify the predictive role of structural and intermediate selected social determinants for different domains of growth and developmental delay, univariable logistic regression was conducted. Variables with a *p*-value less than 0.20 from univariable logistic regression models were entered to multivariable model using the stepwise method. The analysis included five domains of developmental delay and two domains of growth, so seven sets of multivariable logistic regression were conducted to identify predictors of each domain. The significance value for multivariable logistic regression was set at *p* < 0.05.

## Results

A total of 1800 individuals participated in present study with 1750 providing complete responses to all the questions (for regression analysis). Missing numbers for each variable is reported in the respective tables. Because the missing data were at random and less than 5% [36], it did not affect the study results. Demographic characteristics of participants are provided in Table 1. Prevalence of developmental delays varied from 4.28% for personal and social domain to 8% for the problem-solving domain. Low weight growth was the most frequent delay (13.56%) among participants’ children. Table 2 presents the prevalence of growth and developmental delays among participants’ children. The predictive role of selected social determinants for different domains of growth and developmental delays via uni-variable and multivariable logistic regression analysis are provided in Tables 1 and 3.

**Table 1:** Summarized characteristics of participants (*N* = 1750) and Results of univariable logistic regression analysis (OR) to identify probable predictors of growth and developmental delays among participants’ children

Variables		Range		Mean (SD*) or *N* (%)	Communication	Growth motor	Fine motor	Personal & social	Problem-solving	Weight	Height
					OR (*p*)**	OR (*p*)	OR (*p*)	OR (*p*)	OR (*p*)	OR (*p*)	OR (*p*)
Mother’s age (years)		16–49		30.61 (6.88)	1.06 (< 0.001)	1.06 (< 0.001)	0.99 (0.42)	1.05 (0.003)	1.00 (0.46)	1.00 (0.95)	1.04 (0.98)
Father’s age (years)		21–63		36.03 (7.05)	1.05 (< 0.001)	1.06 (< 0.001)	0.96 (0.28)	1.04 (0.005)	1.01 (0.31)	1.00 (0.67)	1.00 (0.63)
Child’s age (months)		3–60		23.81 (18.40)	1.03 (< 0.001)	1.04 (< 0.001)	1.00 (0.18)	1.04 (< 0.001)	1.01 (0.005)	1.00 (0.47)	1.01 (0.99)
Perceived social support	Family	4–20		16.14 (2.65)	0.93 (0.02)	0.93 (0.03)	0.96 (0.25)	0.96 (0.31)	0.99 (0.84)	1.02 (0.43)	1.01 (0.85)
	Friends	4–20		13.74 (3.74)	0.91 (< 0.001)	0.95 (0.05)	0.98 (0.40)	0.98 (0.43)	0.99 (0.52)	1.01 (0.45)	0.94 (0.99)
	Significant others	4–20		16.20 (2.53)	0.90 (0.002)	0.94 (0.09)	1.04 (0.30)	1.00 (0.91)	1.03 (0.48)	0.99 (0.66)	0.91 (0.99)
		Categories		N (%)							
Father’s education		Under high school diploma		287 (15.9)	RG***	RG	RG	RG	RG	RG	RG
		High school diploma		717 (39.85)	0.67 (0.12)	1.13 (0.66)	0.72 (0.20)	1.46 (0.26)	0.78 (0.29)	1.21 (0.38)	1.20 (0.60)
		University degree		796 (44.35)	0.55 (0.02)	0.47 (0.01)	0.80 (0.33)	0.65 (0.24)	0.68 (0.11)	1.48 (0.70)	1.01 (0.98)
Mother’s education		Under high school diploma		321 (17.8)	RG	RG	RG	RG	RG	RG	RG
		High school diploma		655 (36.4)	0.70 (0.14)	0.95 (0.85)	0.61 (0.05)	0.87 (0.63)	0.92 (0.72)	1.49 (0.06)	1.44 (0.29)
		University degree		824 (45.8)	0.53 (0.01)	0.43 (0.003)	0.60 (0.03)	0.48 (0.02)	0.72 (0.16)	1.41 (0.10)	1.16 (0.66)
Father’s job status		Unemployed		22 (1.22)	RG	RG	RG	RG	RG	RG	RG
		Employed		1776 (98.67)	0.14 (< 0.001)	0.38 (0.12)	0.45 (0.21)	0.19 (0.004)	0.29 (0.02)	3.31 (0.24)	0.00 (0.99)
		Missing		1							
Mother’s job status		Housewife		1545 (85.83)	RG	RG	RG	RG	RG	RG	RG
		Employed		251 (13.94)	0.90 (0.71)	1.05 (0.86)	1.31 (0.27)	0.51 (0.12)	1.06 (0.83)	0.64 (0.05)	0.86 (0.65)
		Missing		4							
Father’s smoking		No		1666 (92.6)	RG	RG	RG	RG	RG	RG	RG
		Yes		133 (7.39)	2.09 (0.01)	2.03 (0.02)	1.85 (0.04)	1.92 (0.06)	1.99 (0.009)	1.14 (0.61)	2.06 (0.03)
		Missing		1							
Place of residency		Town		1376 (76.6)	RG	RG	RG	RG	RG	RG	RG
		Village		424 (23.4)	0.32 (< 0.001)	0.23 (< 0.001)	0.67 (0.11)	0.22 (0.001)	0.77 (0.24)	0.89 (0.50)	0.91 (0.73)
Healthcare insurance status		No healthcare insurance		82 (4.6)	RG	RG	RG	RG	RG	RG	RG
		Have healthcare insurance		1718 (95.4)	1.37 (0.54)	1.19 (0.74)	0.65 (0.27)	1.19 (0.78)	0.80 (0.55)	0.75 (0.34)	0.32 (0.01)
Child gender		Boy		802 (44.6)	RG	RG	RG	RG	RG	RG	RG
		Girl		998 (55.4)	0.72 (0.91)	1.06 (0.78)	0.87 (0.45)	0.59 (0.03)	0.77 (0.12)	1.04 (0.77)	1.06 (0.43)
Living with both parents		No		20 (1.1)	RG	RG	RG	RG	RG	RG	RG
		Yes		1780 (98.9)	0.00 (0.99)	0.00 (0.99)	1.37 (0.76)	0.00 (0.99)	0.78 (0.74)	0.29 (0.01)	0.43 (0.27)
Number of children		1		943 (52.4)	1.45 (0.004)	1.09 (0.54)	0.84 (0.23)	1.23 (0.20)	1.11 (0.41)	1.07 (0.84)	1.45 (0.76)
		2		711 (39.5)							
		3 ≤		146 (8.1)							
Present child’s birth order		1		1076 (59.8)	1.23 (0.16)	0.90 (0.55)	0.78 (0.15)	0.62 (0.04)	1.07 (0.63)	1.06 (0.53)	1.76 (0.87)
		2		580 (32.2)							
		3 ≤		144 (8.1)							
Perceived family economic status		Poor		295 (16.39)	RG	RG	RG	RG	RG	RG	RG
		Fair		1254 (69.67)	0.45 (< 0.001)	0.41 (< 0.001)	1.55 (0.14)	0.58 (0.05)	1.07 (0.79)	0.73 (0.08)	0.56 (0.03)
		Good		238 (13.22)	0.71 (0.27)	0.49 (0.04)	1.45 (0.33)	0.57 (0.18)	0.79 (0.50)	1.04 (0.87)	0.52 (0.11)
		Missing		13							
House ownership		Rental		534 (29.7)	RG	RG	RG	RG	RG	RG	RG
		Owned		1255 (69.72)	0.66 (0.04)	0.68 (0.07)	0.73 (0.11)	0.65 (0.08)	0.83 (0.31)	0.87 (0.33)	0.53 (0.01)
		Missing		11							
Pregnancy complication		No		1590 (88.33)	RG	RG	RG	RG	RG	RG	RG
		Yes		180 (10)	0.23 (0.01)	0.69 (0.57)	0.59 (0.19)	0.39 (0.12)	0.26 (0.008)	1.76 (0.01)	1.31 (0.44)
		Missing		30							
Data collection time		Before COVID-19 pandemic		544 (30.2)	RG	RG	RG	RG	RG	RG	RG
		During COVID-19 pandemic		1256 (69.8)	0.71 (0.09)	0.82 (0.35)	0.72 (0.10)	0.81 (0.37)	0.64 (0.01)	0.85 (0.27)	0.49 (0.01)
Maternal general health		Somatic symptoms	Abnormal	716 (39.78)	1.27 (0.22)	1.02 (0.92)	0.76 (0.18)	1.12 (0.65)	0.84 (0.32)	0.93 (0.58)	0.85 (0.49)
			Normal	1068 (59.33)	RG	RG	RG	RG	RG	RG	RG
			missing	16							
		Anxiety and sleeplessness	Abnormal	952 (52.89)	1.02 (0.91)	0.83 (0.36)	0.80 (0.24)	1.21 (0.43)	0.99 (0.94)	1.17 (0.26)	0.61 (0.95)
			Normal	828 (46)	RG	RG	RG	RG	RG	RG	RG
			Missing	20							
		Social dysfunction	Abnormal	334 (18.56)	1.21 (0.47)	0.99 (0.96)	1.09 (0.74)	1.03 (0.93)	1.20 (0.43)	1.23 (0.27)	1.37 (0.32)
			Normal	1443 (80.17)							
			Missing	23							
		Severe depression	Abnormal	640 (35.56)	1.63 (0.02)	1.22 (0.37)	0.91 (0.61)	1.17 (0.53)	1.01 (0.96	1.19 (0.24)	1.42 (0.16)
			Normal	1145 (63.61)	RG	RG	RG	RG	RG	RG	RG
			missing	15							
Household food security		Food secure		1233 (68.9)	RG	RG	RG	RG	RG	RG	RG
		Food insecure		556 (31.1)	1.95 (< 0.001)	1.57 (0.03)	1.26 (0.25)	1.36 (0.20)	0.93 (0.69)	1.21 (0.19)	1.69 (0.02)
		Missing		11							

* SD: standard deviation, ** OR: odds ratio, *p* = *p*-value, *** RG: reference group

N.B. In each domain variable with *p*-value less than 0.2 were selected to enter in multivariable logistic regression model.

**Table 2:** Prevalence of growth and developmental disorders among participants’ children (*N* = 1750)

	Subscales	Point estimated prevalence (%)	95% CI of prevalence*
Developmental disorder	Communication	6.5	5.36; 7.64
	Growth motor	5.72	4.65; 6.80
	Fine motor	6.72	5.56; 7.88
	Personal and social	4.28	3.34; 5.21
	Problem-solving	8.00	6.75; 9.26
Growth disorder	Weight	13.56	11.97; 15.14
	Height	4.66	3.68; 5.63

*95% confidence interval (CI) was calculated based on binomial exact test

**Table 3:** Results of multivariable logistic regression analysis to identify predictors of growth and developmental delays among participants’ children (*N* = 1750)

Developmental delay	Predictors		OR*	95% C.I. OR **	*p*-value	Variance
Communication^1^	Father’s job status of employed vs. unemployed		0.24	0.09; 0.65	0.005	13.8%
	Father’s education of diploma vs. under diploma		0.55	0.35; 0.88	0.012	
	Child’s age		1.04	1.03; 1.05	< 0.001	
	Experience of pregnancy complication		0.24	0.08; 0.78	0.018	
	Father’s cigarette smoking		2.05	1.08; 3.90	0.028	
	Perceived social support from friends		0.92	0.88; 0.97	0.002	
Growth motor^2^	Father’s cigarette smoking		1.76	0.92; 3.37	0.09	10%
	Father’s age		1.06	1.01; 1.11	0.03	
	Child’s age		1.03	1.02; 1.05	< 0.001	
	Mother’s age		0.94	0.89; 1.00	0.05	
	Place of residency (village vs. town)		0.48	0.21; 1.12	0.09	
Fine motor^3^	House ownership (owned vs. rental)		0.66	0.44; 1.00	0.05	1.8%
	Experience of pregnancy complication		0.52	0.22; 1.20	0.13	
	Mother’s education (vs. under diploma)	High school diploma	0.55	0.32; 0.92	0.02	
		Academic degree	0.56	0.34; 0.92	0.02	
Personal & social^4^	Family’s economic status (fair vs. poor)		1.89	1.04; 3.43	0.04	15.1%
	Child’s age		1.06	1.04; 1.07	< 0.001	
	Birth order		0.53	0.33; 0.87	0.01	
	Father’s job status (employed vs. unemployed)		0.15	0.05; 0.50	0.002	
Problem-solving^5^	Father’s smoking		1.66	0.95; 2.90	0.07	2.5%
	Child’s age		1.01	1.00; 1.02	0.02	
	Father job status (employed vs. unemployed)		0.31	0.11; 0.86	0.02	
	Data collection during vs. before COVID-19 pandemic		0.71	0.49; 1.04	0.08	
Weight growth^6^	Pregnancy complication		1.64	1.09; 2.45	0.02	2.2%
	Family’s economic status (fair vs. poor)		0.72	0.54; 0.96	0.03	
	Mother’s job status (employed vs. housewife)		0.68	0.44; 1.07	0.10	
	Living with both parents		0.25	0.09; 0.68	0.006	
Height growth^7^	Household food insecurity vs. security		1.62	1.02; 2.56	0.04	4.3%
	House ownership (owned vs. rental)		0.62	0.39; 0.99	0.05	
	Data collection during COVID-19 pandemic		0.55	0.34; 0.87	0.01	
	Having healthcare insurance		0.44	0.20; 0.95	0.04	

* OR: odds ratio, ** 95% C.I. OR: 95% confidence interval of odds ratio

^1^ Variable(s) entered in Step 1: father’s and mother’s age, father’s job, place of residency, house ownership, family economic stat, father’s and mothers’ education status, child’s age, children number, birth order, experiencing pregnancy complication, data gathered in covid time, father’s smoking, food security, family, friends and others’ social support, maternal depression status.

^2^ Variable(s) entered in Step 1: father’s and mother’s age, father’s job, place of residency, house ownership, family economic stat, father’s and mothers’ education status, child’s age, father’s smoking, food security, family, friends and others’ social support.

^3^ Variable(s) entered in Step 1: family economic stat, fathers and mothers’ education status, place of residency, house ownership, child’s age, birth order, experiencing pregnancy complication, data gathered in covid time, fathers’ smoking, maternal somatic symptoms status.

^4^ Variable(s) entered in Step 1: fathers and mothers’ age, father’s and mothers’ job, place of residency, father’s education ststus, family economic stat, house ownership, child’s age, children no, birth order, child’s gender, experiencing pregnancy complication, father’s smoking, food security

^5^ Variable(s) entered in Step 1: father’s job, child’s age, child’s gender, data gathered in covid time, father’s smoking, fathers and mothers’ education status

^6^ Variable(s) entered in Step 1: mothers’ education status, mother’s job, family economic stat, experiencing pregnancy complication, living with both parents, food security

^7^ Variable(s) entered in Step 1:having insurance, house ownership, family economic stat, data gathered in covid time, father’s smoking, food security

Communication delays were almost twice higher among children whose fathers smoked (*OR: 2.05, 95% CI:1.08; 3.90, p = 0.03*), and 4% higher by each month increase in the child’s age (*OR: 1.04, 95% CI: 1.03; 1.05, p < 0.001*). The risk of communication delays was 76% lower among children whose mothers experienced pregnancy complications (*OR: 0.24, 95% CI: 0.07; 0.78, p = 0.02*), 76% lower among children whose fathers were employed (vs. unemployed, *OR: 0.24, 95% CI: 0.09; 0.65, p = 0.005*), 45% lower among children whose father’s education status was high school diploma (vs. under diploma, *OR: 0.55, 95% CI: 0.35; 0.88, p = 0.01*), and 8% lower among children by each score increase in their mothers perceived social support from friends (*OR: 0.92, 95% CI: 0.88; 0.97, p = 0.002*).

Gross motor delays increased 6% among children by each year increase in their fathers’ age (*OR: 1.06, 95% CI: 1.01; 1.11, p = 0.03*), was 3% higher by each month increase in child’s age (*OR: 1.03, 95% CI: 1.02; 1.05, p < 0.001*), and 76% higher among children whose fathers smoked (*OR: 1.76, 95% CI: 0.92; 3.37, p = 0.09*). They were decreased by 52% among children whose families lived in a village (vs. town, *OR: 0.48, 95% CI: 0.21; 1.12, p = 0.09*) and decreased 9% among children by each year increase in their mothers’ age (*OR: 0.94, 95% CI: 0.89; 1.00, p = 0.05*).

Fine motor delays were 34% lower among children whose families owned their house (*OR: 0.66, 95% CI: 0.55; 1.00, p = 0.05*), 45% lower among mothers with education status of high school diploma (*OR: 0.55, 95% CI: 0.32; 0.92, p = 0.02*) and university degree (*OR: 0.56, 95% CI: 0.34; 0.92, p = 0.02*) vs. under diploma group.

Personal and social delays were significantly increased among children whose families had fair (vs. poor) economic status (*OR = 1.89, 95% CI: 1.04; 3.43, p = 0.04*). It also increased 6% by each month increase in the child’s age (*OR: 1.06, 95% CI: 1.04; 1.07 p < 0.001*). When birth order of children increased, personal and social delays decreased 47% (*OR: 0.53, 95% CI: 0.33; 0.87, p = 0.01*) (i.e., first born children were more likely to have delay problems in this domain compared to children born after other children). This developmental delay was 85% lower among children when their fathers were employed (vs. unemployed, *OR: 0.15, 95% CI: 0.05; 0.50 p = 0.002*).

Problem-solving delays were 66% higher among children whose fathers smoked cigarettes (*OR: 1.76, 95% CI: 0.95; 2.90, p = 0.07*), and increased 1% by each month increase in the child’ age (*OR: 1.01, 95% CI: 1.00; 1.02, p = 0.02*). Problem-solving delay was 69% lower among children whose fathers were employed (vs. unemployed, *OR: 0.31, 95% CI: 0.11; 0.86, p = 0.02*).

Weight growth delays were 64% higher among children whose mothers experienced pregnancy complications (*OR: 1.64, 95% CI: 1.09; 2.45, p = 0.02*). They were 28% lower among children whose families had fair economic status (*OR: 0.72, 95% CI: 0.54; 0.96, p = 0.03*), 32% lower among children whose mothers were employed (vs. housewife, *OR: 0.68, 95% CI: 0.44; 1.07, p = 0.10*), and 75% lower when the child lived with both parents (*OR: 0.25, 95% CI: 0.09; 0.68, p = 0.006*).

Height growth delays were 62% higher among children living households that had food insecurity (*OR: 1.62, 95% CI:1.02; 2.56, p = 0.04*). They were 38% lower among children who lived in a family-owned house (vs. rental, *OR: 0.62, 95% CI: 0.39; 0.99, p = 0.05*), 45% lower when the data were collected during the COVID-19 pandemic (*OR: 0.55, 95% CI: 0.34; 0.87, p = 0.01*), and 56% lower among children whose family had healthcare insurance (*OR: 0.44, 95% CI: 0.20; 0.95, p = 0.04*).

## Discussion

The present study investigated the prevalence and social determinants of growth and developmental delays among children under five years of age. The main findings are provided and discussed below.

In the present study, the prevalence of developmental problems in the five areas ranged from 4.28% for personal and social delays to 8% for problem-solving delays. In a previous Iranian study (in 2014), the prevalence of these developmental delays was similar. In that study, the prevalence of developmental delays was 3.69% (social-personal delay) to 4.31% (fine motor delay) in a nationally representative children of 11,000 children aged 4–60 months [21]. The prevalence of developmental delays among 422 children aged 6–12 months from North West of Iran (in 2017) varied from 0.9% (for gross motor delay) 7.1% (for communication delay) [23]. In the other study from northwest of Iran (in 2021), the prevalence of undetected developmental delay varied from 1.63% (communication delay) to 3.58% (social-personal delay) among 615 children aged 36–60 months [22]. However, there are differences in prevalence of delays among different domains which might be due to different socio-economic and cultural conditions in different parts of the country. In the present study the most prevalent developmental delay was observed in problem-solving, while communication developmental delays were the most prevalent in previous studies [37–39]. The difference might be due to factors such as different age ranges of included children, sampling issues, living environment, health status of children, and socioeconomic characteristics of parents.

In the present study, the prevalence of weight and height growth delays were 13.56% and 4.66% among children aged under five years. The prevalence of children’s growth delay in previous studies has varied among different samples. In Iran (where the present study was carried out), different prevalences of growth delay had been reported from 3.69% to 18.8% [21–23], which is consistent with findings in present study. According to a recent geographical information system-based study of the worldwide pattern of malnutrition, the prevalence of children aged under five years with stunted growth and being underweight in African and Asian countries (especially in the Middle East) was higher compared to rest of the world [40]. Previous studies of weight growth delay among children have reported a prevalence of 15.4% in Sudan (2014) among children aged under five years [41], 13% in Ghana (2015) among children aged under five years [42], 35.1% in Indonesia (Jakarta) (2019) among children aged under five years [43], 25.1% in India (2019) among children aged 10–18 years (2019) [44], 34% among children aged under five years in India (South Delhi) (2020) [45]. The same studies also examined height growth delay and reported a prevalence of 24.9% in Sudan [41], 28% in Ghana [42], 20.9% in Indonesia [43], 32.2% in India [44], and 42.6% in India (South Delhi) [45]. Therefore, African and Asian countries are among the most vulnerable regions for children’s growth and development delays compared to the rest of the world [46], Iran has a lower prevalence of growth delays compared to other Asian and African countries. This difference might be due to extensive network of primary healthcare provision via comprehensive health centers in Iran which provide a valuable opportunity to screen the children’s growth status and provision of timely interventions. The different prevalence of growth and developmental delays might be due to their multifaceted nature and is one of the reasons that the present study investigated some of the social determinants of growth and developmental delays.

In present study, communication, gross motor, and problem-solving delays were higher among children whose fathers smoked cigarettes. Based on animal and human studies, exposure to secondhand smoking might negatively affect neurodevelopment and expression of receptors in the hippocampus [47, 48], inducing oxidative stress in the brain [49], indirect neuronal damage [50] with lasting impairment on cognitive functioning [51]. In the early years of life, brain development is quick and there is a high capacity for change. This time of brain development is fundamental time for lifelong health and wellbeing [52]. Therefore, it is reasonable that exposure to secondhand smoking in childhood could affect different aspects of children’s development [53]. A systematic review showed association of childhood exposure to parental smoking with midlife cognitive function [53]. Therefore, early childhood is a critical period and they should be protected from environmental threats including secondhand smoke exposure.

Father’s employment and education status was associated with communication, personal and social, and problem-solving delays in present study. Also in present study, fine motor delays were lower among children whose families owned their own house and among children with more educated mothers. This is consistent with previous evidence because parental educational status, their occupation, and family income are significant predictors of children’s developmental outcomes [54]. Family socioeconomic status is considered a key factor in child development [55]. The association between higher parental socioeconomic status with lower developmental delays might be better parental interaction with their child. Recent evidence has reported a positive association between early father-infant play and positive social, emotional, and cognitive outcomes [56].

Communication, gross motor, and personal and social delays increased with child’s age in present study. Early identification of children with (or at-risk of) developmental problems is important for intervention and prevention strategies. Identification of developmental delays are not easy and might not be detected until the child is older [57]. Based on current evidence, early motor development is associated with later communication development in infancy [57–59]. In this regard, the results of the present study are consistent with previous studies, showing that identification of some developmental delays are time-dependent and will be detected in older age. Theoretically, child development is affected by various factors including genetic and environmental factors [60], bodily structures, the personal characteristics of the child, and the environmental upbringing that facilitates trial and error experiences [57]. Therefore, some genetic and environmental factors may be considered to have same influence on the emergence of some developmental delays as the association between motor and communication development [57–59]. Consequently, longitudinal studies are needed to investigate the most important factors in the emergence of developmental delays.

In present study, the risk of communication delays was lower among children whose mothers experienced pregnancy complications, and lower among children whose mothers perceived higher social support from friends. The association of high risk pregnancy with developmental delays is not consistent through the literature. Some previous studies reported no association between high risk pregnancy and occurrence of developmental delays [61], while some reported significant higher delay in only some aspects such as fine motor delays [62]. Among gestational complications, those which are uncontrolled or detected late might disturb brain development and consequently affect the child’s development [63]. In the present study, lower risk of communication delays among mothers who experienced pregnancy complications might be due to their more cautious parenting behaviors and more mother-infant interaction which provides a nurturing environment to lessen further problems for their children. Nurturing care is defined as a stable environment, sensitive to children’s health and nutritional needs, protective from different threats, with various age- appropriate opportunities, with sufficient interactions with children that are developmentally stimulating [64]. Another finding observed in present study was lower communication delay among children whose mothers perceived higher social support from friends. Having more social support can provide an environment with more interactions and learning opportunity for early child development [65, 66].

In present study, gross motor delays were higher among children by each year increase in their father’s age. They were lower among children increase in their mothers’ age. Based on a recent systematic review, there is no consistent evidence regarding the association of parents’ age and prevalence of gross motor delays [67]. Therefore, further studies are needed to investigate the association of parent’s age with different aspects of children’s development.

In present study, personal and social delay was significantly increased among children whose families had fair (vs. poor) economic status. This is inconsistent with previous evidence that family income (i.e., socioeconomic status) is reported to be a significant predictor of children’s developmental outcomes [54, 55]. This inconsistency might be due to difference in the family process. Family process is a critical mediator of the effects of economic hardship on children’s social adjustment. The elevated perceptions of economic pressure can indirectly affect parental psychological well-being and parenting behavior [68]. Among families with fair economic status, mothers are more likely to be employed and parents spend more time engaged in job-related tasks. Therefore, they might spend less time with their child. Lower parents-child interaction might lead to increased personal and social delays [68]. On the other hand, having a more interactive family environment and supporting family processes provide more learning opportunities for children and lower children’s social delay [64] which is consistent with another finding of the present study that with increased birth order of children, personal and social delays decreased.

In the present study, weight growth delay was higher among children whose mothers had experienced pregnancy complications, and lower among children (i) whose families had fair economic status, (ii) whose mothers were employed, and (iii) who lived with both parents. Previous research has consistently shown that significant factors associated with children’s weight and height growth delays include mothers’ problems during pregnancy [42, 69], family socioeconomic conditions [41, 70–74], household income [72, 75], mother’s employment [70] and living with both parents [76]. In the present study, height growth delays were higher among children from households with food insecurity, and lower among children whose family owned their own house and had healthcare insurance. They were also lower when the data were collected during the COVID-19 pandemic. Similarly, previous research has shown that significant factors associated with children’s stunted growth include food insecurity [75, 77], and not having a permanent place of residence [78]. Overall, reviews of the existing literature regarding growth delays among children aged under five years indicate that the most important socio-economic contributing factors are (i) parents’ (and especially mothers’) low education, (ii) low household income, (iii) food insecurity, and (iv) living in marginalized and deprived areas. Addressing social and economic factors, as well as increasing health access, are important factors that can increase the growth and development of children and reduce child mortality [15, 79–84].

Despite the concerns regarding the negative impact of COVID-19 pandemic as unexpected or the temporal situation on children’s growth and development based on current review papers [85–87], the present study did not find many negative influences of COVID-19 pandemic on children’s growth and development. In the univariable regression analysis, data collected during COVID-19 pandemic was associated with problem-solving domain of development (36% lower during the pandemic) and height growth delays (51% lower during the pandemic). In the multivariable regression models, data collected during pandemic was significant predictor for only height growth delays. Height growth disorders were 45% lower based on data collected during the COVID-19 pandemic. It could be that during the COVID-19 pandemic, parents were more attentive to their children’s growth. Also, family members had to spend more time at home together (due to social distancing and home quarantines policies). This may have led to higher quality parent-child interactions. To best of the present authors’ knowledge, no previous studies have examined the growth and development of children at two time points before and during COVID-19 pandemic. In Indonesia, Fitriahadi (2021) et al. assessed the socio-demographic predictors of growth and development of children aged under five years. They found that the predictors of growth and development among children aged under five years during the COVID-19 pandemic were poor maternal education and poor family income [88] which were same predictors as before the pandemic [15, 79–84].

Overall present study investigated the significant social determinants of children’s growth and development delays. Identifying high-risk children might help healthcare providers to develop appropriate interventions including providing nutritional supplements and educational programs to enhance parent-child interactions for families with low economic status. Screening for the social determinants of health to identify at-risk individuals have been introduced in other countries [80–82]. Therefore, the present study’s findings could help in the design and implementation of timely interventions to screen for at-risk of growth and developmental delays among Iranian children aged under five years.

## Strengths and limitations

In the present study, an attempt was made to identify social factors associated with growth and development delays among children aged under five years based on the comprehensive framework proposed by the World Health Organization. A large sample size and the application of a multivariable logistic regression model helped to identify the most significant factors associated with these developmental delays. However, some limitations should be considered in the interpretation of the findings. The recruitment of participants was carried out in comprehensive health centers, so all participants had free access to healthcare services. Therefore, the role of access to healthcare services could not be evaluated with these particular participants. The study design was cross-sectional, which is appropriate for assessing prevalence and associations but limits the ability to establish causality. The number of participants in some subgroups, (such as fathers who smoked cigarettes, drunk alcohol, and used drugs) was low, and which might be under-reported due to social desirability bias. Due to low number of participants in these subgroups, proper analysis could not be conducted, which means these factors need further investigation to confirm the findings. The present study also used convenience sampling in Qazvin health centers. However, by sampling from comprehensive urban health centers, sampling was carried out in all geographic and social areas in Qazvin in order to achieve diversity of socio-economic characteristics. However, data collection using self-report questionnaires might increase the chance of recall bias. Finally, the data were only collected in Qazvin, therefore the sample was not representative all children aged under five years in Iran.

## Conclusion

In the present study, the most important social determinants of growth and developmental delays among Iranian children below the age of five years were determined. The most prevalent developmental problem among children aged below five years was in the problem-solving domain. There were different predictors of growth and developmental delay problems among Iranian children aged under five years including fathers’ cigarette smoking, families’ economic status, and household food insecurity as well as history of mothers’ pregnancy complications. These findings show the importance of the need for early screening and detection of children’s growth and developmental delays for timely intervention. Moreover, the different risk factors of growth and developmental delays necessitate their screening for the social determinants of health to identify at-risk individuals, which have been introduced in other countries. Therefore, the present study’s findings could help in the design and implementation of timely interventions to screen for at-risk of growth and developmental delays among Iranian children aged under five years.

## Data Availability

Data and materials will be provided upon reasonable request to the corresponding author.

## Abbreviations

ASQ: Ages and Stages Questionnaire
HFIAS: Household Food Insecurity Access Scale
GHQ: General Health Questionnaire
MSPSS: Multidimensional Scale of Perceived Social Support
OR: Odds ratio
SD: Standard deviation
WHO: World Health Organization
